# Sustainable collaboration on complex problems: a “who” not a “what” challenge

**DOI:** 10.3389/frma.2023.1224030

**Published:** 2023-09-21

**Authors:** Josie Gibson

**Affiliations:** CatalystFX and The Catalyst Network, Richmond, VIC, Australia

**Keywords:** collaboration, collective leadership, systems change, networks, First Nations knowledge systems

## Abstract

Despite decades of collective efforts and millions of dollars of cross-sector investment, collaborations created to address wicked problems—complex issues that span industries and sectors whose root causes are unclear—have had mixed success. The wicked problems terrain is tribal and competitive. It is contested by proponents of competing collective change and innovation tools and methodologies, advocates of different leadership approaches and, in recent years, big business champions who claim private enterprise is the most effective driver of solutions. This perspective article argues that while all these elements deserve attention, the primary focus of many collaborations reflects a Western scientific bias toward “what” and “how” questions—governance, processes, activities, metrics and outcomes—at the expense of the “who” component: the human relationships, or relational infrastructure, required to build and sustain effective collective efforts. This is crucial given the grueling realities of complex multi-year initiatives. This article explores the tension between this bias and the need to develop robust relational networks through skilful collective leadership, as reflected in numerous First Nations knowledge practices. We discuss leadership as a both an individual and a collective capability and highlight the need for better understanding of its significant role in anchoring, shaping and guiding effective system-based efforts that achieve positive impact.

## 1. Introduction

The litany of complex challenges we face is long and depressing. Problems range from climate change and biodiversity loss to geopolitical turmoil, income inequality, youth mental health, mass job losses and social breakdown, not to mention the cumulative impacts of a global pandemic. The roots of such issues date back years, raising an obvious question: Why, after so much attention, effort and investment, have expensive solutions failed to make headway on these challenges? Is it the leaders themselves, or how we approach these challenges, that ultimately derails our collective ambitions?

This paper relates my 10-year investigation into these questions. My inquiry has involved deep dives into academic research on leadership, collaboration, project management and complex problem-solving, interviews with researchers, and client engagements and field experiments with *de jour* design and delivery methodologies and other aspects of multi-stakeholder collaborations. The more I've learned, the more I'm aware of how little I know. My biases and blind spots are numerous. Yet large parts of this scenario don't make sense. With the impact investment market alone topping US$1 trillion, there's no shortage of capital trying to fund potential solutions (Hand et al., 2022).

## 2. Collective leadership is the key

I now believe the type of leadership on these initiatives is the primary determinant of success or failure. Most approaches reflect a Western bias that ignores the realities of human nature. Every day that bias costs us dearly in sub-standard outcomes. I've witnessed well-meaning people in senior leadership roles who are ill-equipped to handle the levels of complexity and ambiguity and the onerous and unrealistic governance and delivery requirements of multi-stakeholder collaborations.

Whether we like it or not, people in positions of leadership, authority or influence shape the context and guidelines for decisions that inform behaviors, action and impact on such projects. Yet we continue to ignore the critical impact of leadership and default to rigid governance models and processes that don't align with how people actually interact. An ingrained bias for action means project leaders rarely take the time to build sustainable relationships and fully understand the nuances of the operating context. There's little space in which to build genuine collective intelligence.

What I've discovered isn't new. Pioneering thinkers, researchers and community development practitioners have trod this path many times before, trialing promising theories and novel approaches that failed to gain mainstream adoption. Often it's a case of being too far ahead of the curve, or not connecting across disciplines.

Times, however, are changing. An exciting development is the increasing body of First Nations scholarship that highlights alternative problem-solving models based on ancient principles grounded in relationships and collective contribution. This cutting-edge work often involves emerging technologies as both a tool and a research focus (Kelleher, 2023). Relational systems thinking scholar Melanie Goodchild suggests that new possibilities lie in the ethical space between Western and non-Western epistemologies (Goodchild, 2021). I share her optimism about what's possible.

By weaving in complementary aspects of these differing worldviews, or ways of experiencing the world, I believe the time is ripe to create new collective leadership approaches that provide the contextual adaptability, shared commitment and momentum that complex endeavors demand. As megaprojects researcher Bent Flyvbjerg notes, “projects don't so much *go* wrong as they *start* wrong” (Flyvbjerg and Gardner, 2023). So let's throw out the old frame and start afresh.

## 3. Wisdom principles guide action

My process of inquiry went like this: a deep dive into leadership, psychology and related realms, exploring the role and influence of individuals in complex systems. I then followed the premise that our failure to crack open these big issues was a system-level design and/or delivery issue. I turned to process design, project management, governance, metrics and related elements, looking at research and field work involving different methodologies and approaches. I also experimented within the changemaker group I run, The Catalyst Network.

Eventually I came full circle, drawn back to how individuals and complex adaptive systems interact. This interface offers many personal anecodotes about collective leadership in complex environments, but I'm surprised to find little field data and academic research on the topic.

Collaboration practitioners like Ed Morrison acknowledge the value of age-old principles through their emphasis on conversation, “the world's oldest social technology,” as the starting point for multi-year projects (Morrison et al., 2019). Only in the last decade, though, has the spotlight been turned specifically on First Nations knowledge systems and practices, with research exploring wicked problems through the lens of wisdom traditions.^1^ These practices suggest we have the prevailing problem-solving model inside out, an argument that will resonate with skilled collaborators.

Australian academic Tyson Yunkaporta's landmark book, *Sand Talk*, provided a rare glimpse into the cosmologies, kinship laws and relational principles at the core of Australian Aboriginal cultures. Yunakporta interviewed Elders and wisdom-keepers from different Aboriginal nations and identified common ways of interaction very different to those of many non-Aboriginal people. “I referred to it as spirit, heart, head and hands,” Yunkaporta wrote. “Mumma Doris knew it as Respect, Connect, Reflect, Direct” (Yunkaporta, 2019).

“*The first step of Respect is aligned with values and protocols of introduction, setting rules and boundaries. This is the work of your spirit, your gut. The second step, Connect, is about establishing strong relationships and routines of exchange that are equal for all involved. Your way of being is your way of relating, because all things exist in relationship to other things. This is the work of your heart. The third step, Reflect, is about thinking as part of the group and collectively establishing a shared body of knowledge to inform what you will do. This is the work of the head. The final step, Direct, is about acting on their shared knowledge in ways that are negotiated. This is the work of the hands” (ibid)*.

As a model for more effective project management, Mumma Doris' description is illuminating. She told Yunkaporta that non-Aboriginal people “seemed to work through the same steps but in reverse.” In other words, a typical big project about to go off the rails progresses as: Direct (impose and fail), Reflect (urgently problem-solve), belatedly Connect with community, and finally, Respect the collective knowledge that was there all along.

These principles are common to collective cultures around the world. The inference is that leaders who focus on building a strong understanding of the relational landscape to underpin activities are more likely to deliver what's promised, in a way that's aligned with all parties' expectations. This is not stakeholder engagement or planning in a Western sense, but deep listening and sensemaking with the express purpose of creating a shared view that reflects the expectations and commitment of those involved. It may take more time upfront to build this foundation, but it pays dividends in delivery.

## 4. Wicked problems are stubborn

Anyone who has worked in a large bureaucracy or long-term project knows collaboration can be frustrating and exhausting. Working with others to deliver shared outcomes isn't easy. Collaborative efforts by their nature cross boundaries, budgets and fiefdoms so they are constantly vulnerable to derailment from time and resource constraints, strategy shifts, and sabotage from cultural and structural barriers.

First defined in the context of urban planning, wicked problems describe complex challenges with no clear boundaries or causes, multiple dimensions and no obvious solutions (Rittel and Webber, 1973). Formidable firepower has been directed at such issues over many decades. There have been some successes but the common narrative is one of expensive failure.

“Cocreation” became a buzzword in the early 2000s, with an increasing focus on collaborations involving not-for-profits, community organizations, philanthropists and big business, often through corporate social responsibility (CSR) activities. A study by scholar-practitioners Joanna Levvitt Cea and Jess Rimington found few efforts delivered the desired results:

“*We were surprised to find that many of the big names in cocreation—including those speaking the loudest about seemingly cutting-edge practices like “collective impact,” “crowdsourcing,” and “design thinking”—were not actually significantly departing from the status quo, particularly when it came to generating a shift in power, voice, and ownership. Instead, breakout actors tend to be on the fringes of their fields.”* (Cea and Rimington, 2017)

The United Nations Sustainable Development Goals (SDGs) are one example of a high-profile multilateral commitment to deal with big, interconnected challenges. Environmental governance specialist Benjamin Cashore argues that despite huge amounts of money, time and effort invested in the SDGs, issues like collective climate action haven't delivered. In fact, Cashore asserts that solutions to address some SDGs have actually worsened other SDG outcomes (Cashore, 2022).

The business end of the wicked problems spectrum doesn't fuel optimism, either. In recent years governments have been spending vast sums of taxpayer dollars on engineering megaprojects to address infrastructure needs such as hospitals, airports, railways, roads and major sporting facilities. Defined as US$1 billion or more, the design and delivery of megaprojects is generally undertaken by private contractors over many years. Research shows major projects are plagued by scope creep, litigation, reputational damage and massive time and cost blowouts (Changali et al., 2015).

My experience in the construction arena shows that little has changed. Despite extensive evidence pointing to chronic leadership and culture issues, studies show that process and governance issues continue to trump people and culture on these staggeringly complex projects (Pau et al., 2016). And the price we pay gets higher and higher (Flyvbjerg and Gardner, 2023).

## 5. Complex vs. complicated

While working on major projects I was eager to learn more about how people manage to thrive in complex environments. Around me I saw technical experts struggling while a small number of peers took scope changes, setbacks and uncertainty in their stride.

**Figure 1:**
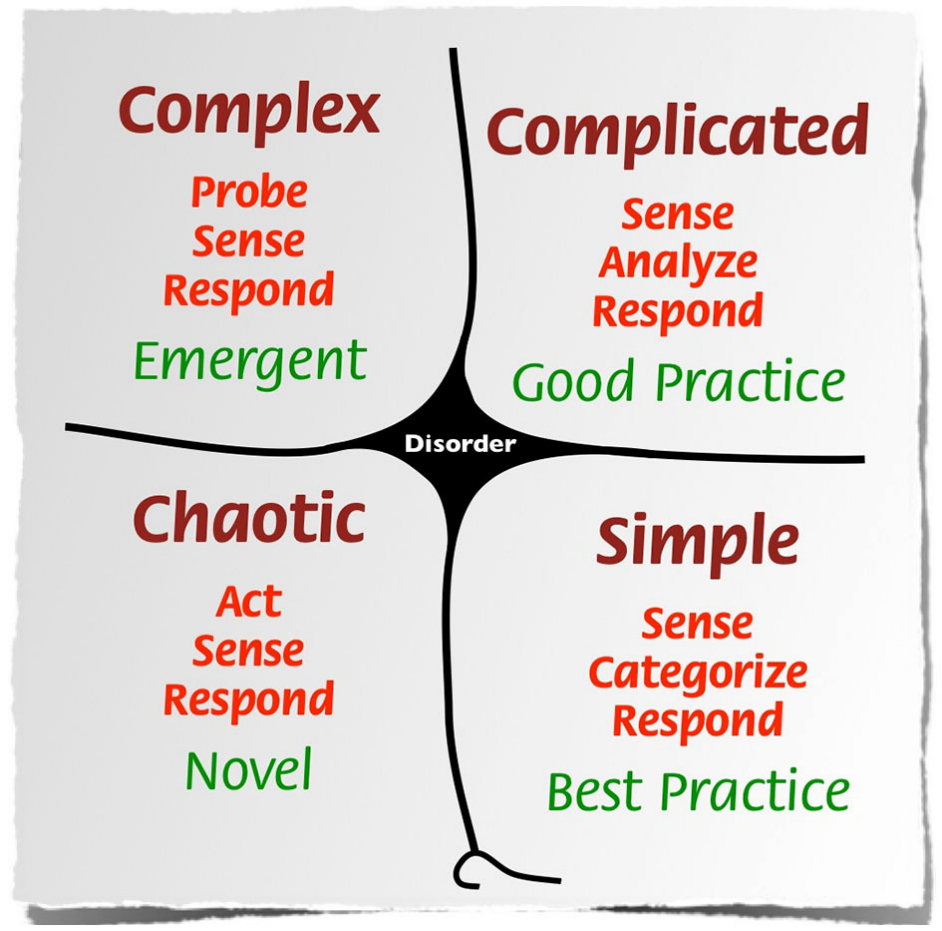
Cynefin framework developed by Snowden and Boone (2007, November). Source: Wikipedia Commons.

Venturing into the realm of complexity science, the Cynefin framework struck an immediate chord. Dave Snowden's visual model helps people differentiate between domains and the behaviors best suited to each (Snowden and Boone (2007, November)). I was interested in the different mindsets required for the complicated and complex domains, shown in Figure 1.

These domains overlap confusingly on major projects, in policy arenas, and in community initiatives, often leading to mismatches of people, management styles, tools, methodologies, decision-making models and expectations.

In the complicated domain there is a clear relationship between cause and effect. Complicated issues are best addressed by expert analysis of multiple options to determine the optimal choice. There is no clear cause and effect in the complex domain. It is a state of fluidity and emergence where, Snowden writes, “instead of attempting to impose a course of action, leaders must patiently allow the path forward to reveal itself.”

This resonated strongly with what I was learning about how different individuals experience the world, and what that means in major problem-solving contexts.

## 6. Not all leaders are equal

Skilled collaborators accept complex terrains, and the people in it, for what they are. They work to see the whole, not just the parts, scan for patterns and signals of change, and understand the intersecting relationships that underpin and influence any project, initiative, workplace or community. This is often called systems thinking (Arnold and Wade, 2015). Management thinkers like Peter Senge have applied systems thinking concepts to leadership at an individual and a collective level (Senge, 1990). Like Mumma Doris, Senge emphasizes the need to cultivate diverse voices to address system-level challenges (Senge et al., 2015).

While this seems self-evident, it's not common practice on typical complex undertakings, where the overriding initial focus is usually on technical expertise or financial models. The balance between people and process is badly skewed. To understand this disconnect, I delved into the arena of adult development and found independent scholar Susanne Cook-Greuter's seminal work on ego development (Cook-Greuter, 1985). I wanted to understand how different people reacted to different levels of volatility and complexity.

Constructive-developmental theory is a branch of psychology that tracks how adults develop through increasingly complex “stages,” create meaning and act based on how they experience the world. Action logic frameworks are a widely used development tool drawing from this research. From a practitioner viewpoint, while there are differences across the body of research, there is strong alignment in the work of leading researchers in the field.

Many scholars have built on on Cook-Greuter's work. Leadership specialist Barrett Brown used the adult development lens to study a group of sustainability leaders in action, in the context of what are now known as the SDGs. His was the first leadership study to have such a large population of very rare late-stage leaders and look at how they engage in complex change initiatives (Brown, 2012).

While acknowledging that the design of sustainability initiatives was a key success factor, Brown argued that a crucial influence on the design was the worldview of the designer. “Leaders with more complex meaning-making systems have access to enhanced and new capacities that others do not,” he said. “These expanded and novel abilities can be—and may *need* to be—leveraged in order to optimally respond to the tremendously complex global challenges we face” (ibid).

For his study, Brown drew upon the action logic work of Cook-Greuter and William Torbert, who applied a leadership lens to the framework (Rooke and Torbert, (2005, April)). The eight most prevalent action logics in Torbert's model are shown in Table 1.

**Table 1:** The eight most prevalent action logics of Torbert's framework.

**Action logic**	**Main focus**	**Characteristics**	**Strengths as org. member**	**Source of power**	**% of US adult population (*N* = 4,510)**
Opportunist (needs rule impulses)	Own immediate needs, opportunity, self-protection	*Wins any way possible*. Self-oriented; manipulative; “might makes right”	Good in emergencies and sales opportunities	Coercive (unilaterally) e.g., executive authority	4.3
Diplomat (norms rule needs)	Socially expected behavior, approval	*Avoids overt conflict*. Wants to belong; obeys group norms; rarely rocks the boat	Good as supportive glue within an office; helps bring people together	Diplomatic e.g., persuasive power, peer power	11.3
Expert (craft logic rules norms)	Expertise, procedure and efficiency	*Rules by logic and expertise*. Seeks rational efficiency	Good as an individual contributor	Logistical e.g., knowledge-based or authoritative power	36.5
Achiever (system effectiveness rules craft logic)	Delivery of results, effectiveness, goals, success within system	*Meets strategic goals*. Effectively achieves goals through teams; juggles managerial duties and market demands	Well-suited to managerial roles; action and goal oriented	Coordinating (coordinating the previous three sources of power)	29.7
Individualist (relativism rules single-system logic)	Self in relationship to system, interaction with system	*Interweaves competing personal and company action logics*. Creates unique structures to resolve gaps between strategy and performance	Effective in venture and consulting roles	Confronting; used to deconstruct others' frames or worldviews	11.3
Strategist (most valuable principles rule relativism)	Linking theory and principles with practice; dynamic systems interactions	*Generates organizational and personal transformations*. Exercises the power of mutual inquiry, vigilance and vulnerability for both the short and long term	Effective as a transfomational leader	Integrative (consciously transformative)	4.9
Alchemist (deep processes and inter-systemic rule principles)	Interplay of awareness, thought, action and effects; transforming self and others	*Generates social transformations*. Integrates material, spiritual and societal transformations	Good at leading society-wide transformations	Shamanism (through presence)	1.5
Ironist	Being; experience moment to moment arising out of consciousness	[Currently under research] *Institutionalizes developmental processes* through “liberating disciplines.” Holds cosmic or universal perspective; visionary	[Currently under research] Create the conditions for deep development of individuals and collectives	[Currently under research] Unitive	0.5

Reprinted by permission of (Brown, 2012).

Barrett and others' research shows that leaders with sophisticated meaning-making systems seek out diverse perspectives that may not be immediately congruent but that could prove critical to the overall picture. They are able to connect and make links between unlikely players. Building trust and collective decision-making capability in these conditions is part of their practice.

While this is statistically a small group, my personal experience is that such leaders have a profound impact because the network of relationships they help to shape at the outset is robust enough to withstand the peaks and troughs of prolonged delivery. They adapt rapidly to shifts in contexts and can help others reframe, overcome blind spots and embrace new ways of doing things to ensure people adapt and progress continues.

An obvious action for organizations and institutions arising from this is to invest in identifying individuals with these characteristics and practices and support them in developing new relational models for complex work. Research shows most individuals can move through action logic stages with the right coaching and support, developing their tolerance of increasingly complex challenges. However, this would take courage as it's counter to traditional leadership development frameworks.

Western concepts of leadership have become heavily commoditised in the past decade or so. *Forbes* estimates US$166 billion is spent each year on leadership development in the US alone, with questionable results (Gurdian et al., 2014). Critics like Harvard leadership lecturer Barbara Kellerman contend that “the rise of leadership as an object of our collective fascination has coincided precisely with the decline of leadership in our collective estimation” (Kellerman, 2012).

Senge, Cook-Greuter and others point out that most leadership theory and change research is filtered through a Western (white) lens. In contrast, there is strong resonance in how Barrett's sustainability leaders approached their challenges and the sequencing of collective cultures. The focus is on getting to know and understand key relationships, identifying the shared vision, wisdom and knowledge resources available, then proceeding to action based on that collective view.

## 7. Conclusion

When it comes to how we tackle the big issues we face as a society, we have the equation the wrong way around: the “who” is more important than the “what” and “how” in shaping effective problem-solving approaches. It is of course a balancing act depending on context and circumstances, but research suggests that some individuals are better at leading and maintaining complex collaborations than others, and that should be acknowledged and factored in at the outset of any project. More broadly, First Nations knowledge systems and practices offer powerful alternative ways to shape and tackle complex issues in a manner that delivers for all parties.

I hope my observations generate useful debate and further research and cross-disciplinary and cross-sector experimentation. If we are more discerning upfront about those we trust to find solutions or deliver what society needs, perhaps we might finally break free of our problem addiction and achieve traction on these big issues.

## Data availability statement

The original contributions presented in the study are included in the article/supplementary material, further inquiries can be directed to the corresponding author.

## Author contributions

The author confirms being the sole contributor of this work and has approved it for publication.

## Footnotes

^1^Indigenous Knowledge Institute (University of Melbourne, Australia), Indigenous Knowledges Systems Lab (Deakin University, Australia), Turtle Island Institute (Canada) et al.
